# Imported Chikungunya Virus Strains, Taiwan, 2006–2014

**DOI:** 10.3201/eid2211.160404

**Published:** 2016-11

**Authors:** Cheng-Fen Yang, Chien-Ling Su, Tung-Chien Hsu, Shu-Fen Chang, Chien-Chou Lin, Jason C. Huang, Pei-Yun Shu

**Affiliations:** Centers for Disease Control, Taipei, Taiwan (C.-F. Yang, C.-L. Su, T.-C. Hsu, S.-F. Chang, C.-C. Lin, P.-Y. Shu);; National Yang Ming University, Taipei (C.-F. Yang, J.C. Huang)

**Keywords:** chikungunya virus, viruses, chikungunya, epidemiology, imported cases, vector-borne infections, zoonoses, Taiwan

## Abstract

We identified 78 imported chikungunya cases in Taiwan during 2006–2014. Sixty-six (84.6%) cases were initially suspected to be dengue, which indicates the necessity for laboratory diagnostics in differentiation between dengue and chikungunya. Results also emphasize the need for active surveillance of febrile illness at points of entry.

Chikungunya is a mosquitoborne viral disease characterized by symptoms, including fever, rash, myalgia, and polyarthralgia that are usually self-limiting and rarely fatal; however, arthralgia might persist (*1*). The causative agent of this disease is chikungunya virus (CHIKV), which belongs to the family *Togaviridae*, genus *Alphavirus* (*2*). Genotypes of CHIKV include West African, East/Central/South African (ECSA), and Asian.

Since 2000, CHIKV has caused unanticipated, large outbreaks in Africa and Asia and become a major public health concern (*3*). In late 2013, CHIKV reached the Americas and caused several explosive outbreaks (*4*). CHIKV is predominantly transmitted by *Aedes aegypti* and *Ae. albopictus* mosquitoes (*1*). Although chikungunya is not endemic to Taiwan, *Aedes* mosquitoes are found throughout Taiwan (*5*). Infected travelers with high viremia who arrive or return from disease-endemic areas could lead to local transmission and outbreaks in Taiwan.

## The Study

To reduce the risk for importation and subsequent spread of arboviruses in Taiwan, active (e.g., fever screening at airports and expanded screening for contact with confirmed cases) and passive (e.g., clinician- or hospital-based) surveillance systems were implemented by the central and local health departments. Serum samples from persons with suspected cases were submitted to the Taiwan Centers for Disease Control (Taipei, Taiwan) for confirmation of arboviral infection.

Because symptoms of different arboviral infections might be similar, we performed multiplex real-time reverse transcription PCR (RT-PCR) with flavivirus consensus primers, alphavirus consensus primers, and virus-specific primers and CHIKV and dengue virus IgM and IgG ELISAs for samples collected from all persons with suspected arboviral infections (*6*,*7*). CHIKV infection was confirmed by detection of CHIKV RNA, isolation of CHIKV, seroconversion, or > 4-fold increase in IgM or IgG titers against CHIKV in paired serum samples (*8*). An imported case of chikungunya was defined as disease in an infected patient who had been traveling abroad >2 weeks before onset of illness.

A total of 78 laboratory-confirmed chikungunya patients who satisfied the definition of an imported case were identified during 2006–2014. Among these patients, 6 persons had suspected CHIKV infections and 4 had suspected CHIKV and dengue virus infections. Two persons were identified though contacts of persons with confirmed cases of chikungunya. The remaining 66 persons were initially reported as having suspected dengue infections.

We determined frequencies of imported chikungunya cases by year and country of origin (Table 1). The first imported case was detected in November of 2006. Since then, imported cases have been detected every year in Taiwan. The most frequently reported countries of origin were Indonesia, the Philippines, and Malaysia.

**Table 1:** Annual number of imported cases of chikungunya, by country of origin and genotype distribution of chikungunya virus strains from imported cases, Taiwan, 2006–2014*

Country	Year									Total
	2006	2007	2008	2009	2010	2011	2012	2013	2014	
Indonesia	0	3 (0,3,0)	4 (0,4,0)	4 (1,3,0)	12 (5,5,2)	0	1 (0,1,0)	17 (0,9,8)	5 (0, 5, 0)	46 (6,30,10)
Philippines	0	0	0	0	0	1 (0, 1, 0)	3 (0, 0, 3)	8 (0,0,8)	1 (0, 1, 0)	13 (0,2,11)
Malaysia	0	0	3 (3,0,0)	2 (2,0,0)	1 (0,0,1)	0	0	0	0	6 (5,0,1)
Thailand	0	0	0	2 (2,0,0)	0	0	0	2 (1,0,1)	0	4 (3,0,1)
Singapore	1 (1,0,0)	0	0	1 (1,0,0)	0	0	0	2 (0,1,1)	0	4 (2,1,1)
Bangladesh	0	0	1 (1,0,0)	0	0	0	0	0	0	1 (1,0,0)
India	0	0	1 (1,0,0)	0	0	0	0	0	0	1 (1,0,0)
Myanmar	0	0	0	0	0	1 (0,0,1)	0	0	0	1 (0,0,1)
Cambodia	0	0	0	0	0	0	1 (0,0,1)	0	0	1 (0,0,1)
Guatemala	0	0	0	0	0	0	0	0	1 (0,0,1)	1 (0,0,1)
Total	1 (1,0,0)	3 (0,3,0)	9 (5,4,0)	9 (6,3,0)	13 (5,5,3)	2 (0,1,1)	5 (0,1,4)	29 (1,10,18)	7 (0,6,1)	78 (18,33,27)

*Values in parentheses indicate number of East/Central/South African genotypes, Asian genotypes, and unidentified chikungunya virus strains, respectively, identified in each country per year.

We found no clear seasonal difference in importation frequency among chikungunya cases (Technical Appendix Figure). Most (57/78, 73%) cases were identified by screening for fever at airports, and 86% (67/78) were reported <7 days of illness onset. Main purposes of travel were business trips of foreign workers, tourism, and family visits (Table 2).

**Table 2:** Characteristics of 78 patients with chikungunya, Taiwan, 2006–2014

Characteristic	No. (%)
Case reporting system	
Fever screening at airports	57 (73.0)
Expanded screening for contacts of confirmed case-patients	2 (3.0)
Clinician- or hospital-based	19 (24.3)
Age group, y	
<20	9 (11.5)
20–39	43 (55.1)
40–59	17 (22.0)
>60	9 (11.5)
Onset day	
<7	67 (86.0)
>7	11 (14.1)
Laboratory confirmation test	
Viral RNA or virus isolation	59 (76.0)
Serologic analysis	19 (24.3)
Travel purpose	
Foreign labor	26 (33.3)
Tourism	25 (32.0)
Family visit	14 (18.0)
Business trip	8 (10.3)
Other	5 (6.4)

Nucleotide sequences of complete structural protein genes C-E3-E2-6K-E1 (capsid–envelope–6K) of 56 imported CHIKV strains were analyzed. A phylogenetic tree was generated by using the maximal-likelihood method and a general time-reversible model in MEGA version 6 (http://www.megasoftware.net/) (*9*).

Imported CHIKV strains were divided into 2 genotypes: ECSA and Asian. The ECSA genotype was the most common genotype of imported CHIKV strains before 2010; strains with this genotype were mainly from Bangladesh, Malaysia, India, and Thailand. The Asian genotype was the most common genotype after 2011; strains with this genotype were mainly from Indonesia, the Philippines, and Singapore (Table 1). A phylogenetic tree of Asian genotype strains was constructed (Figure, panel A).

**Figure:**
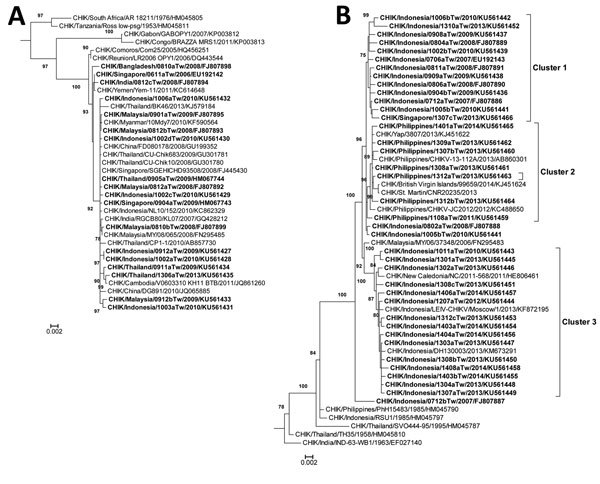
Phylogenetic analysis of chikungunya virus (CHIKV) isolates from imported cases of chikungunya in Taiwan, 2006–2014. Trees show genetic relationships of the East/Central/South African genotype (A) and Asian genotype (B) of CHIKV isolates; clusters are shown in panel B. Trees were generated by using nucleotide sequences (3,747 bp) of complete structural protein genes C-E3-E2-6K-E1 (capsid–envelope–6K) of CHIKV strains. Sequences obtained in this study are indicated in bold. Viruses are identified by virus/country/strain/year of isolation/GenBank accession no. Strains reported in this study were submitted to GenBank under accession nos. FJ807886–FJ807895, FJ807898, FJ807899, EU192142, EU192143, HM067743, HM067744, and KU561427–KU561466. Analysis was performed by using MEGA version 6 software (http://www.megasoftware.net/) and the maximal-likelihood method (general time-reversible model). Bootstrap support values >75 are shown (1,000 replicates) along branches. Scale bars indicate nucleotide substitutions per site.

Most of the imported strains could be grouped into 3 clusters. Cluster 1 contains CHIKV strains imported from Indonesia during 2007–2013 and Singapore during 2013. Cluster 2 contains CHIKV strains imported from the Philippines during 2011–2014. These strains are closely related to those isolated from Yap and St. Martin during 2013 and from the British Virgin Islands during 2014. Cluster 3 contains strains imported from Indonesia during 2010–2014. These data suggest that strains from Indonesia have shifted from cluster 1 to cluster 3 in recent years. A phylogenetic tree of CHIKV strains with the ECSA genotype was constructed (Figure, panel B). A total of 18 imported CHIKV strains from India and Southeast Asia also had the ECSA genotype. All of these strains were grouped together and are closely related to strains from islands in the Indian Ocean.

Although most isolates from Indonesia had the Asian genotype, there were 6 E1–226V variants imported from Indonesia during 2009–2010 that had the ECSA genotype. Relevant amino acid changes in complete structural proteins of imported CHIKV strains are shown in the Technical Appendix Table.

## Conclusions

A total of 78 imported chikungunya cases were identified in Taiwan during 2006–2014. With the exception of 1 imported chikungunya case from Guatemala, countries of origin for all imported cases were in southern and Southeast Asia. The location of these countries reflects the frequency of air travel between Taiwan and these countries and might also reflect the frequency and intensity of chikungunya outbreaks in these countries during the same period.

CHIKV has caused major outbreaks in southern and Southeast Asia since late 2005 (*10**–**14*). Our results are consistent with the ECSA genotype being prevalent in Singapore, Indonesia, Malaysia and Thailand during 2006– 2010. The Asian genotype was prevalent in Indonesia and the Philippines during 2007–2014. We found that the E1-226V variants of the ECSA genotype were imported from Indonesia in 2009 (*15*). Our data are concordant with the chikungunya epidemic status in Southeast Asia, including Indonesia and the Philippines, in recent years (*12*,*13*). However, the low number of imported chikungunya cases in Taiwan does not allow for a meaningful statistical analysis.

Because symptoms of many arboviral infections are similar, surveillance strategies in Taiwan for different arboviruses are also similar. Most of the confirmed chikungunya cases were initially reported as suspected dengue cases, which indicates that it is necessary to perform diagnostic tests for chikungunya and dengue in suspected cases.

During 2006–2014, a total of 36,150 suspected cases of arboviral infections were initially screened for CHIKV by RT-PCR and IgM/IgG ELISA (using the first blood sample). Most case-patients were in the acute phase of the disease. Among the remaining suspected case-patients, convalescent-phase serum samples were collected from 320 patients for testing by ELISA. Results suggested that the positive rate for CHIKV (78/36,150, 0.22%) was low among persons with suspected cases of arboviral infection. In addition, no indigenous chikungunya cases have been identified in this study.

Most confirmatory testing was performed by using RT-PCR. However, this testing could lead to a lower rate of laboratory-confirmed chikungunya cases because there was limited serologic testing completed for paired serum samples. In addition, the population not captured (e.g., those with subclinical or nonacute infections) could result in an underestimation of the number of imported chikungunya cases.

To prevent spread of arbovirus diseases, well-organized integrated disease and vector surveillance systems must be properly implemented and executed. Detection of imported chikungunya cases by active and passive surveillance at an early stage is needed to implement early response activities and reduce risk for local transmission.

Technical Appendix Additional information on imported chikungunya virus strains, Taiwan, 2006–2014.
