# Supplementary material for: Imported Chikungunya Virus Strains, Taiwan, 2006–2014
Source: Emerg Infect Dis. 2016 Nov;22(11):1981–4. doi: 10.3201/eid2211.160404 (PMC5088036; doi:10.3201/eid2211.160404)
Supplement: Technical Appendix — Additional information on imported chikungunya virus strains, Taiwan, 2006–2014. [file 16-0404-Techapp-s1.pdf]

Article DOI: http://dx.doi.org/10.3201/eid2211.160404

Imported Chikungunya Virus Strains, Taiwan, 2006–2014

Technical Appendix

Technical Appendix Table. Relevant amino acid changes in structural proteins of 57 imported chikungunya virus strains, Taiwan, 2006–2014*

	Protein, polypeptide position, protein position																
	C	C	E3	E2	E2	E2	E2	E2	E2	E2	6K	E1	E1	E1	E1	E1	E1
	81	93	280	571	573	577	598	632	645	696	785	813	1035	1093	1130	1158	1206
Strain	81	93	19	246	248	252	273	307	320	371	37	4	226	284	321	349	397
ECSA genotype																	
CHIK/India/0812cTw/2008	T	A	Q	A	L	**K**	N	Q	T	V	R	V	A	E	A	N	L
CHIK/Singapore/0611aTw/2006	T	A	Q	A	L	**K**	N	Q	T	V	R	V	A	E	A	N	L
CHIK/Singapore/0904aTw/2009	T	A	Q	A	L	Q	N	Q	T	V	R	V	**V**	E	A	N	L
CHIK/Bangladesh/0810aTw/2008	T	A	Q	A	L	**K**	N	Q	**A**	V	R	**A**	**V**	E	A	**D**	L
CHIK/Malaysia/0810bTw/2008	T	A	Q	A	L	Q	N	Q	**I**	V	R	V	**V**	E	A	N	L
CHIK/Malaysia/0812aTw/2008	T	A	Q	A	L	Q	N	Q	T	V	R	V	**V**	E	A	N	L
CHIK/Malaysia/0812bTw/2008	T	A	Q	A	L	Q	N	Q	T	V	R	V	**V**	E	A	N	L
CHIK/Malaysia/0901aTw/2009	T	A	Q	A	L	Q	N	Q	T	V	R	V	**V**	E	A	N	L
CHIK/Malaysia/0912bTw/2009	T	A	Q	A	L	Q	N	Q	T	V	R	V	**V**	E	A	N	L
CHIK/Thailand/0905aTw/2009	T	A	Q	A	L	Q	N	Q	T	V	R	V	**V**	E	A	N	L
CHIK/Thailand/0911aTw/2009	T	A	Q	A	L	Q	**I**	Q	T	V	R	V	**V**	E	A	N	L
CHIK/Thailand/1306aTw/2013	T	A	Q	A	L	Q	**I**	Q	T	V	R	V	**V**	E	A	N	L
CHIK/Indonesia/0912aTw/2009	T	A	Q	A	L	Q	N	Q	T	V	R	V	**V**	E	A	N	L
CHIK/Indonesia/1002aTw/2010	T	A	Q	A	L	Q	N	Q	T	V	R	V	**V**	E	A	N	L
CHIK/Indonesia/1002cTw/2010	T	A	Q	A	L	Q	N	Q	T	V	R	V	**V**	E	A	N	L
CHIK/Indonesia/1002dTw/2010	T	A	Q	A	L	Q	N	Q	T	V	R	V	**V**	E	A	N	L
CHIK/Indonesia/1003aTw/2010	T	A	Q	A	L	Q	N	Q	T	V	R	V	V	E	A	N	L
CHIK/Indonesia/1006aTw/2010	T	A	Q	A	L	Q	N	Q	T	V	R	V	V	E	A	N	L
Asian genotype																	
CHIK/Indonesia/0712bTw/2007	M	V	Q	A	**L**	K	N	Q	T	V	R	V	A	D	A	N	P
CHIK/Indonesia/0802aTw/2008	T	V	Q	A	F	K	N	Q	T	L	R	V	A	D	A	N	P
CHIK/Indonesia/1005bTw/2010	T	V	Q	A	F	K	N	Q	T	L	R	V	A	D	A	N	P
CHIK/Indonesia/0706aTw/2007	T	V	Q	A	F	K	N	Q	T	L	R	V	A	D	A	N	P
CHIK/Indonesia/0712aTw/2007	T	V	Q	A	F	K	N	Q	T	L	R	V	A	D	A	N	P
CHIK/Indonesia/0804aTw/2008	T	V	Q	A	F	K	N	Q	T	L	R	V	A	D	A	N	P
CHIK/Indonesia/0806aTw/2008	T	V	Q	A	F	K	N	Q	T	L	R	V	A	D	A	N	P
CHIK/Indonesia/0811aTw/2008	T	V	Q	A	F	K	N	Q	T	L	R	V	A	D	A	N	P
CHIK/Indonesia/0904bTw/2009	T	V	Q	A	F	K	N	Q	T	L	R	V	A	D	A	N	P
CHIK/Indonesia/0908aTw/2009	T	V	Q	A	F	K	N	Q	T	L	R	V	A	D	A	N	P
CHIK/Indonesia/0909aTw/2009	T	V	Q	A	F	K	N	Q	T	L	R	V	A	D	A	N	P
CHIK/Indonesia/1002bTw/2010	T	V	Q	A	F	K	N	Q	T	L	R	V	A	D	A	N	P
CHIK/Indonesia/1005aTw/2010	T	V	Q	A	F	K	N	Q	T	L	R	V	A	D	A	N	P
CHIK/Indonesia/1006bTw/2010	T	V	Q	**D**	F	K	N	Q	T	L	R	V	A	D	A	N	P
CHIK/Indonesia/1310aTw/2013	T	V	Q	**D**	F	K	N	Q	T	L	R	V	A	D	A	N	P
CHIK/Singapore/1307cTw/2013	T	**I**	Q	A	**S**	K	N	Q	T	L	**K**	V	A	D	A	N	P
CHIK/Philippines/1108aTw/2011	T	V	Q	A	F	K	N	Q	T	L	R	V	A	D	A	N	P
CHIK/Philippines/1307bTw/2013	T	V	**R**	A	F	K	N	Q	T	L	R	V	A	D	A	N	**L**

	Protein, polypeptide position, protein position																
	C	C	E3	E2	E2	E2	E2	E2	E2	E2	6K	E1	E1	E1	E1	E1	E1
	81	93	280	571	573	577	598	632	645	696	785	813	1035	1093	1130	1158	1206
Strain	81	93	19	246	248	252	273	307	320	371	37	4	226	284	321	349	397
CHIK/Philippines/1308aTw/2013	T	V	**R**	A	F	K	N	Q	T	L	R	V	A	D	A	N	**L**
CHIK/Philippines/1309aTw/2013	T	V	**R**	A	F	K	N	Q	T	L	R	V	A	D	A	N	**L**
CHIK/Philippines/1312aTw/2013	**A**	V	**R**	A	F	K	N	Q	T	L	R	V	A	D	A	N	**L**
CHIK/Philippines/1312bTw/2013	T	V	**R**	A	F	K	N	Q	T	L	R	V	A	D	A	N	**L**
CHIK/Philippines/1401aTw/2014	T	V	**R**	A	F	K	N	Q	T	L	R	V	A	D	A	N	**L**
CHIK/Indonesia/1011aTw/2010	M	V	Q	A	S	K	N	Q	T	V	R	V	A	D	**V**	N	P
CHIK/Indonesia/1207aTw/2012	M	V	Q	A	S	K	N	**R**	T	V	R	V	A	D	T	N	P
CHIK/Indonesia/1301aTw/2013	M	V	Q	A	S	K	N	Q	T	V	R	V	A	D	**V**	N	P
CHIK/Indonesia/1302aTw/2013	M	V	Q	A	**A**	K	N	Q	T	V	R	V	A	D	**A**	N	P
CHIK/Indonesia/1303aTw/2013	M	V	Q	A	S	K	N	**R**	T	V	R	V	A	D	**T**	N	P
CHIK/Indonesia/1304aTW/2013	M	V	Q	A	S	K	N	**R**	T	V	R	V	A	D	**T**	N	P
CHIK/Indonesia/1307aTw/2013	M	V	Q	A	S	K	N	**R**	T	V	R	V	A	D	**T**	N	P
CHIK/Indonesia/1308bTw/2013	M	V	Q	A	S	K	N	**R**	T	V	R	V	A	D	**T**	N	P
CHIK/Indonesia/1308cTw/2013	M	V	Q	A	S	K	N	Q	T	V	R	V	A	D	**A**	N	P
CHIK/Indonesia/1312cTw/2013	M	V	Q	A	S	K	N	**R**	T	V	R	V	A	D	**T**	N	P
CHIK/Indonesia/1403aTw/2014	T	V	Q	A	S	K	N	**R**	T	V	R	V	A	D	**T**	N	P
CHIK/Indonesia/1403bTw/2014	M	V	Q	A	S	K	N	**R**	T	V	R	V	A	D	**T**	N	P
CHIK/Indonesia/1404aTw/2014	M	V	Q	A	S	K	N	**R**	T	V	R	V	A	D	**T**	N	P
CHIK/Indonesia/1406aTw/2014	M	V	Q	A	S	K	N	**R**	T	V	R	V	A	D	**A**	N	P
CHIK/Indonesia/1408aTw/2014	M	V	Q	A	S	K	N	**R**	T	V	R	V	A	D	**T**	N	P

*Molecular signatures are indicated in bold. C, capsid; CHIK, chikungunya; E, envelope; ECSA, East/Central/South African.

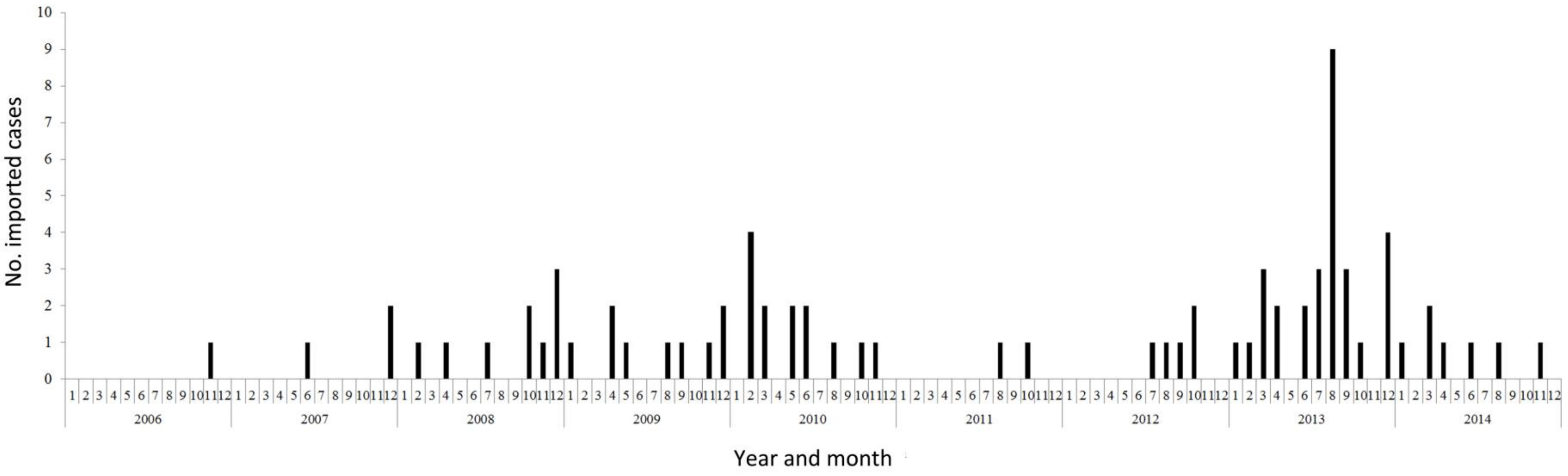

Technical Appendix Figure. Distribution of imported chikungunya cases, by month and year, Taiwan, 2006–2014.
